# Immune classification of osteosarcoma

**DOI:** 10.3934/mbe.2021098

**Published:** 2021-02-22

**Authors:** Trang Le, Sumeyye Su, Leili Shahriyari

**Affiliations:** Department of Mathematics and Statistics, University of Massachusetts Amherst, Amherst, MA MA 01003-9305, USA

**Keywords:** osteosarcoma, immune pattern, gene expression analysis

## Abstract

Tumor immune microenvironment has been shown to be important in predicting the tumor progression and the outcome of treatments. This work aims to identify different immune patterns in osteosarcoma and their clinical characteristics. We use the latest and best performing deconvolution method, CIBERSORTx, to obtain the relative abundance of 22 immune cells. Then we cluster patients based on their estimated immune abundance and study the characteristics of these clusters, along with the relationship between immune infiltration and outcome of patients. We find that abundance of CD8 T cells, NK cells and M1 Macrophages have a positive association with prognosis, while abundance of *γδ* T cells, Mast cells, M0 Macrophages and Dendritic cells have a negative association with prognosis. Accordingly, the cluster with the lowest proportion of CD8 T cells, M1 Macrophages and highest proportion of M0 Macrophages has the worst outcome among clusters. By grouping patients with similar immune patterns, we are also able to suggest treatments that are specific to the tumor microenvironment.

## 1. Introduction

Osteosarcoma is the most common type of malignant bone tumors that appears most frequently in children and young adults. It usually starts in the femur, the tibia and the humerus, and less commonly the skull, the jaw or the pelvis [1]. There are some risk factor such as age, height, gender, and heritable syndromes that predispose to for osteosarcoma, however, the cause of osteosarcomas is still not clear [2].

Although neoadjuvant chemotherapy in treatment of osteosarcoma has shown better results, overall survival of patients with metastasis still remains in low rate in the last three decades [3, 4]. Immunotherapy and targeted therapy have recently shown significant improvements for some malignancies [5, 6]. Therefore, they are increasingly attractive treatment options for ostesarcoma patients. However, they are not effective for many patients and there is no evidence that proves the effectiveness of a specific molecular target [7].

It has been shown in many studies that cancer cells and tumor infiltrating immune cells (TIICs) have an important role in cancer progression and determination of malignant tumor types [8–10]. CD8 T cells, which is an important component of TIICs, has been found to associate with better clinical outcomes and response to immunotherapy in many cancer types [11, 12]. Treatments that includes the antitumor immunocompetence of innate immune cells, which includes NK cells and *γδ* T cells, are found favorable for osteosarcoma tumors [13, 14]. These studies show the importance of finding further biomarkers of tumor cells such as PD-1 and CTLA-4 and of tumor infiltrating immune cells such as T cells and Macrophages to improve new treatment strategies and to choose the best treatment strategy for osteosarcoma patients.

Several computational methods, alternative ways to immunohistochemistry and flow cytometry [15], have been recently developed to derive tumor immune infiltrates using gene expression profiles of bulk tumors. In this study, we use the latest popular deconvolution method [16–20], CIBERSORTx [21], to investigate the immune patterns of tumors and analyze the relationship between immune composition and clinical features of osteosarcoma patients.

There have been many other studies that utilize a deconvolution method to study the tumor microenvironment of osteosarcoma. A number of them use the estimated immune infiltrations calculated using CIBERSORT and/or immune scores calculated using ESTIMATE to find immune-related genes that can predict the prognosis of osteosarcoma [22–25]. Another set of studies find genes with prognostic values by applying Cox model on survival data or performing differentially expressed genes analysis between two groups of interest, and then investigate the relationship between these genes and estimated immune infiltrates [26–30]. Others study the association of immune abundance with clinical information directly [24, 31–34]. Our work falls somewhat into the third category.

Among the studies that directly investigate the relationship between immune infiltrations and clinical information, three of them use TARGET data set [24, 31, 33], one uses data from GSE21257 [32], and one uses data from GSE39058 [34]. Most of these studies use CIBERSORT [24, 32–34], while the other uses ssGSEA and ImmuCellAI along with expression of immune marker genes to get estimated abundance of immune cells [31]. Our study, on the other hand, utilizes the latest and best performing version of deconvolution methods called CIBERSORTx B-mode, and conducts our analyses on both TARGET data set and GSE21257 data set. We also perform K-means clustering using the estimated immune abundance to study the clinical characteristics of different immune patterns in osteosarcoma. Another study has used hierarchical clustering on immune abundance, but they estimate immune abundance using ssGSEA and do not focus on the clinical difference between clusters [35].

## 2. Materials and methods

### 2.1. Data collection and processing

The gene expression data sets in this study are obtained from 2 cohorts: TARGET (cohort 1) and GSE21257 (cohort 2). Cohort 1 includes FPKM normalized RNA-seq data of 88 osetosarcoma patients downloaded from the UCSC Xena web portal and their corresponding clinical data downloaded from the GDC data portal. Cohort 2 includes microarray data and corresponding clinical features of 53 osteosarcoma samples, downloaded from GEO website. Cohort 2’s gene expression data were previously normalized with robust spline normalization before being downloaded.

### 2.2. Relative abundance of immune cells

We calculate the relative frequencies of 22 immune cell types by applying CIBERSORTx [21] B-mode algorithm with immune signature matrix LM22 on gene expression data from both cohort 1 and 2. CIBERSORTx is the latest version of CIBERSORT (Cell type Identification By Estimating Relative Subsets Of RNA Transcripts) [36], where batch correction method Combat has been introduced in the algorithm to reduce the effect of cross-platform variation between gene expression data and signature matrix. There are 2 modes of this algorithm: B-mode and S-mode, whose difference lies in the way batch correction is applied in the algorithm. Our recent study has shown that CIBERSORTx B-mode gives good estimates of immune abundance in both RNA-Seq and microarray data with the use of LM22, and in fact outperforms CIBERSORT and other tumor deconvolution methods [16]. We obtain estimated immune fractions by running the algorithm on their website with 100 permutations. Similar to CIBERSORT, CIBERSORTx outputs a p-value for each deconvolved sample as an indicator of confidence of the results. We use samples with p-value less than 0.05 for analyses in this study.

### 2.3. Identification of immune patterns in osteosarcoma

To calculate the abundance of each cell type, which has several subtypes with a small abundance, we sum the proportions of their subtypes obtained from CIBERSORTx. The abundance of B cells is the summation of naive and memory B cells; NK cell is the summation of resting and activated NK cells; Mast cells is the summation of resting and activated Mast cells; Dendritic cells is the summation of resting and activated Dendritic cells; and CD4 T cells is the summation of follicular helper T cells, regulatory T cells, naive CD4 T cells, resting and activated memory CD4 T cells. We do not combine subtypes of Macrophages because M1 and M2 Macrophages have very different functions and Macrophages make up the majority of immune cells in osteosarcoma.

We then use K-means clustering to identify various immune patterns of osteosarcoma based on the estimated immune cells’ abundance. The number of clusters in K-means is determined using elbow method. A t-SNE visualization of the clusters is also included to see how well K-means algorithm distinguishes between samples with different immune patterns.

### 2.4. Immune scores of osteosarcoma tumors

Immune scores of all samples in cohort 1 and 2 are computed from ESTIMATE algorithm [37]. In order to do so, we run *estimate* package locally from R. We also divide all patients from both cohorts into low immune score and high immune score group using the median immune score as cut-off to study the relationship between immune score and survival outcome.

### 2.5. Statistical analysis

Chi-square test is used to analyze the relationship between categorical variables in this study. We employ Mann-Whitney-Wilcoxon test to detect any significant difference between groups of continuous variables, such as immune fractions, gene expression, age and immune score. Pearson correlation and corresponding p-value are used to study the correlation between different immune infiltrates.

To investigate the impact of immune infiltrates on survival, for each immune cell, we split all patients into high and low abundance group using the median value as cut-off and perform log-rank test to find significant difference in survival between groups. Kaplan-Meier curves are also plotted to visualize the differences between these groups.

All analyses in this study are conducted on all samples in both cohorts 1 and 2, except for metastasis-free survival analysis which is applied only on cohort 2 since cohort 1 does not include data on the time of metastasis development. All statistical analyses and visualizations are carried out in Python.

## 3. Results

### 3.1. The most abundant immune cells in osteosarcoma are Macrophages and CD4 T cells

**Figure 1.**
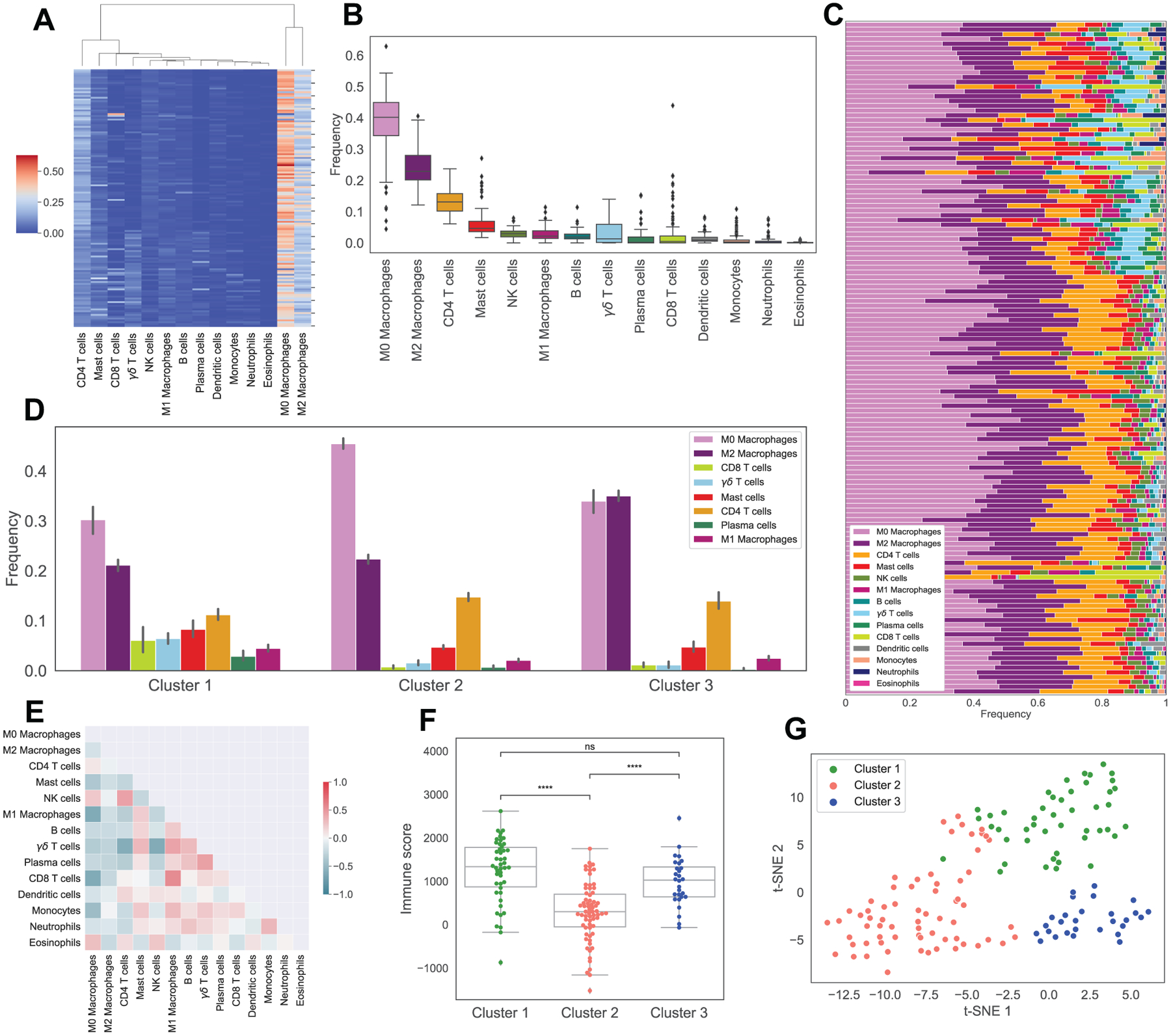
Immune pattern of osteosarcoma. Sub-figure A shows the hierarchical clustering of estimated immune cells’ infiltration. Sub-figure B and C display the boxplot and stacked barchart of these immune cells’ fractions. Sub-figures D shows the average frequencies of immune cells in 3 clusters obtained from K-means clustering. Sub-figure E indicates the correlation map of immune cell frequencies. Sub-figure F displays the boxplot of ESTIMATE immune scores in 3 clusters, with asterisks indicating significant difference from Mann-Whitney-Wilcoxon test (ns: no significance, *: 0.01 < *p* ≤ 0.05, **: 0.001 < *p* ≤ 0.01, ***: 0.0001 < *p* ≤ 0.001, ****: *p* ≤ 0.0001). Sub-figure G shows t-SNE plot of estimated immune abundance, color coded by cluster.

Results of CIBERSORTx B-mode on gene expression profiles of 141 osteosarcoma patients (cohorts 1 and 2) demonstrates that M0 Macrophages is the most frequent immune cell in osteosarcoma tumors with an average of 40% of total immune cells, followed by M2 Macrophages and CD4 T cells (Figure 1B, C). Unsupervised hierarchical clustering of immune cell fractions shows that most abundant cells tend to be clustered together, as is shown in Figure 1A, where M0 and M2 Macrophages are clustered together and then grouped with CD4 T cells and other immune cells. In addition, the most frequent cells also have the highest variation in abundance across osteosarcoma tumors (Figure 1B).

### 3.2. Correlation between immune infiltrates in osteosarcoma

According to the CIBERSORTx B-mode results, abundance of CD8 T cells is negatively correlated with M0 Macrophages and positively correlated with M1 Macrophages with Pearson correlation coefficients of −0.62 and 0.55, with p-values of 4.8e–16 and 1.1e–12, respectively (Figure 1E). The proportion of *γδ* T cells is also significantly negatively correlated with CD4 T cells and NK cells (Pearson coefficients of −0.6 and −0.62, with p-values of 4.3e–15 and 1.8e–16). Interestingly, frequencies of M0 and M1 Macrophages exhibit a negative correlation of −0.61 with p-value 1e–15.

### 3.3. There are 3 immune patterns of osteosarcoma

K-means clustering of immune cell proportions in osteosarcoma tumors indicates the existence of three distinct immune classes (Figure 1D), namely: Cluster 1, which has the highest proportions of CD8 T cells, *γδ* T cells, M1 Macrophages, Mast cells and Plasma cells and the lowest proportion of M0 Macrophages; Cluster 2, in which the percentage of M0 Macrophages is the highest; and Cluster 3, which has the highest percentage of M2 Macrophages. A t-SNE plot of immune cell proportions suggests that K-means clustering algorithm successfully separates osteosarcoma patients with different immune patterns (Figure 1G).

### 3.4. Cluster 2 has the worst survival outcome among all clusters

**Figure 2.**
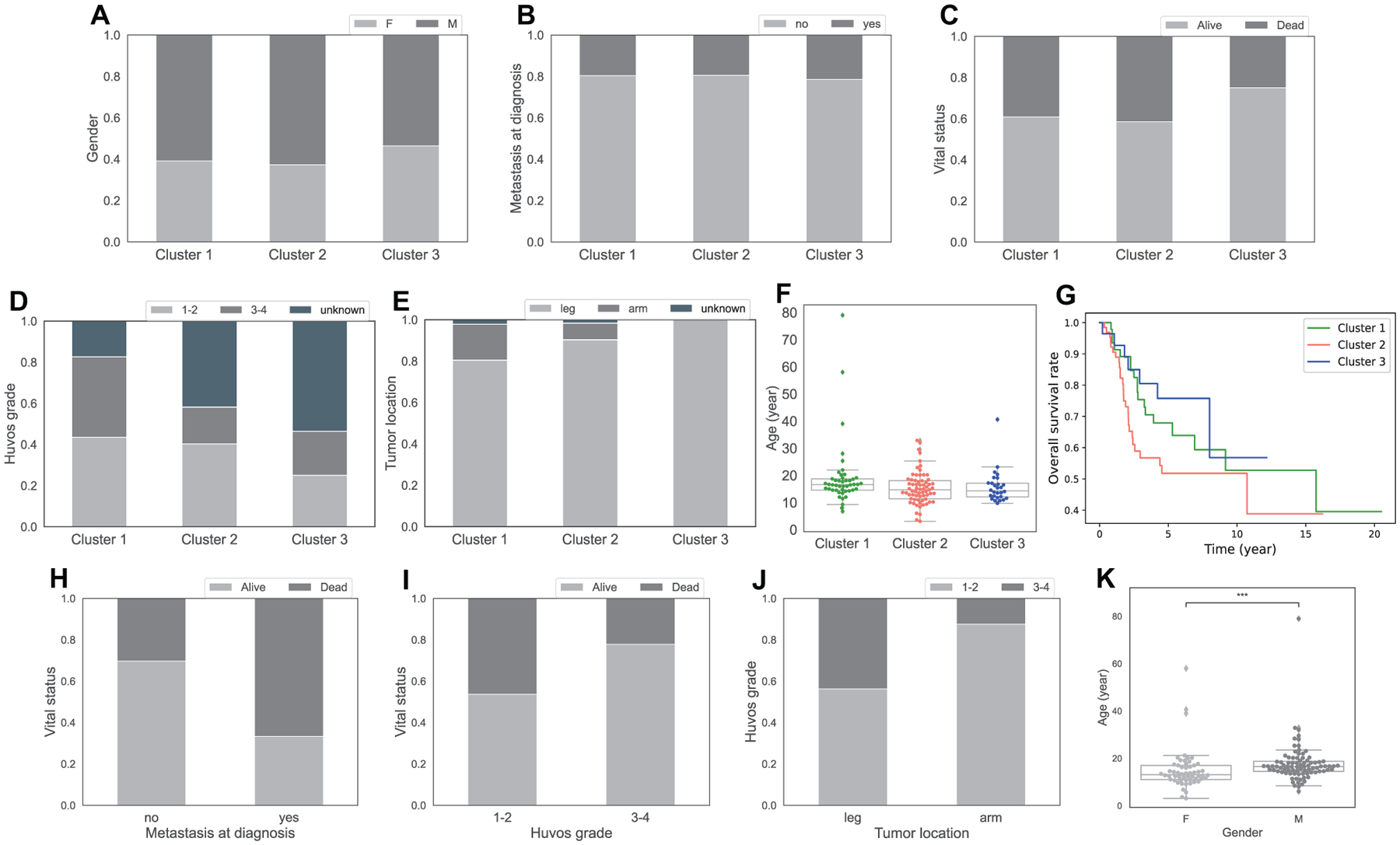
Clinical characteristics of clusters and relationship between clinical features of osteosarcoma. Sub-figure A-E show percentage of patients by gender (A), metastasis at diagnosis (B), vital status at the last time of follow-up (C), Huvos grade (D), primary tumor location (E), in the 3 clusters. Sub-figure F shows a boxplot of patients’ age at diagnosis in each cluster. Sub-figure G displays Kaplan-Meier curves of overall survival across 3 clusters. Sub-figures H-K shows the association between clinical features, H: percentage of alive and dead patients by metastasis at diagnosis, I: percentage of alive and dead patients by Huvos grade, J: percentage of low and high Huvos grade by tumor location, K: boxplot of age at diagnosis by gender, with asterisks indicating significant difference from Mann-Whitney-Wilcoxon test (ns: no significance, *: 0.01 < *p* ≤ 0.05, **: 0.001 < *p* ≤ 0.01, ***: 0.0001 < *p* ≤ 0.001, ****: *p* ≤ 0.0001

While there is no significant difference in gender, age and proportion of metastasis at diagnosis between clusters (Figure 2A, B, F), we observe some differences in survival outcomes among clusters.

Kaplan-Meier curves indicate that cluster 2 has the worst survival probability throughout time out of all clusters (Figure 2G). In addition, cluster 2, along with cluster 1, has higher percentage of dead patients at the last time of follow up than cluster 3 (Figure 2C). Interestingly, cluster 2 also has the lowest immune scores compared to other clusters (Figure 1F).

Cluster 3 appears to have the best outcome among clusters. It has the lowest percentage of dead patients at the last time of follow up among all clusters (Figure 2C), and better survival rate than cluster 2 over time (Figure 2G) with p-value 0.07 from the log-rank test.

Cluster 1, which has the highest amount of CD8 T cells, *γδ* T cells, M1 Macrophages and Mast cells, has slightly better overall survival time than cluster 2 (p = 0.16, Figure 2G). However, cluster 1 seems to have worse outcome than cluster 3 due to its higher percentage of dead patients at the last time of follow up (Figure 2C). It is worth noting that there is no significant difference in the survival rate between cluster 1 and 3 according to the log-rank test (p = 0.5, Figure 2G).

### 3.5. There is a relationship between certain clinical features of osteosarcoma

The Chi-square test and Mann-Whitney-Wilcoxon test show a relationship between metastasis at diagnosis and vital status (p = 0.001, Figure 2H), Huvos grade, which is a grading system to evaluate a patient’s response to chemotherapy based on the percentage of necrosis in the tumor after treatment, and vital status (p = 0.036, Figure 2I), and between gender and age (p = 2.3e–4, Figure 2K) where male patients are older on average. We observe that patients with metastasis at diagnosis have much higher percentage of being dead at the last time of follow up than patients without metastasis (Figure 2H). This makes perfect sense since metastases have been known to associate with late stages of tumor and poor prognosis in many cancers. The other clinical feature with a relation to vital status is Huvos grade. Higher percentage of patients with high Huvos grade (3–4) are alive at the last time of follow up than patients with low Huvos grade (1–2) (Figure 2I), which is reasonable since a high Huvos grade means good response to chemotherapy. Figure 2J suggests that primary osteosarcoma tumors in the arm respond more poorly to chemotherapy than leg tumors, as illustrated by the high proportion of Huvos grade 1–2 in arm tumors. However, it is important to note that primary osteosarcoma tumors happen more often in the leg than in the arm (Figure 2E), and the observation in Figure 2J is based on a small number of arm tumor samples (n = 8), thus the relationship between tumor location and Huvos grade is not considered significant by the Chi-square test (p = 0.18).

### 3.6. Immune score does not relate to vital status directly, but does relate to survival probability over time

**Figure 3.**
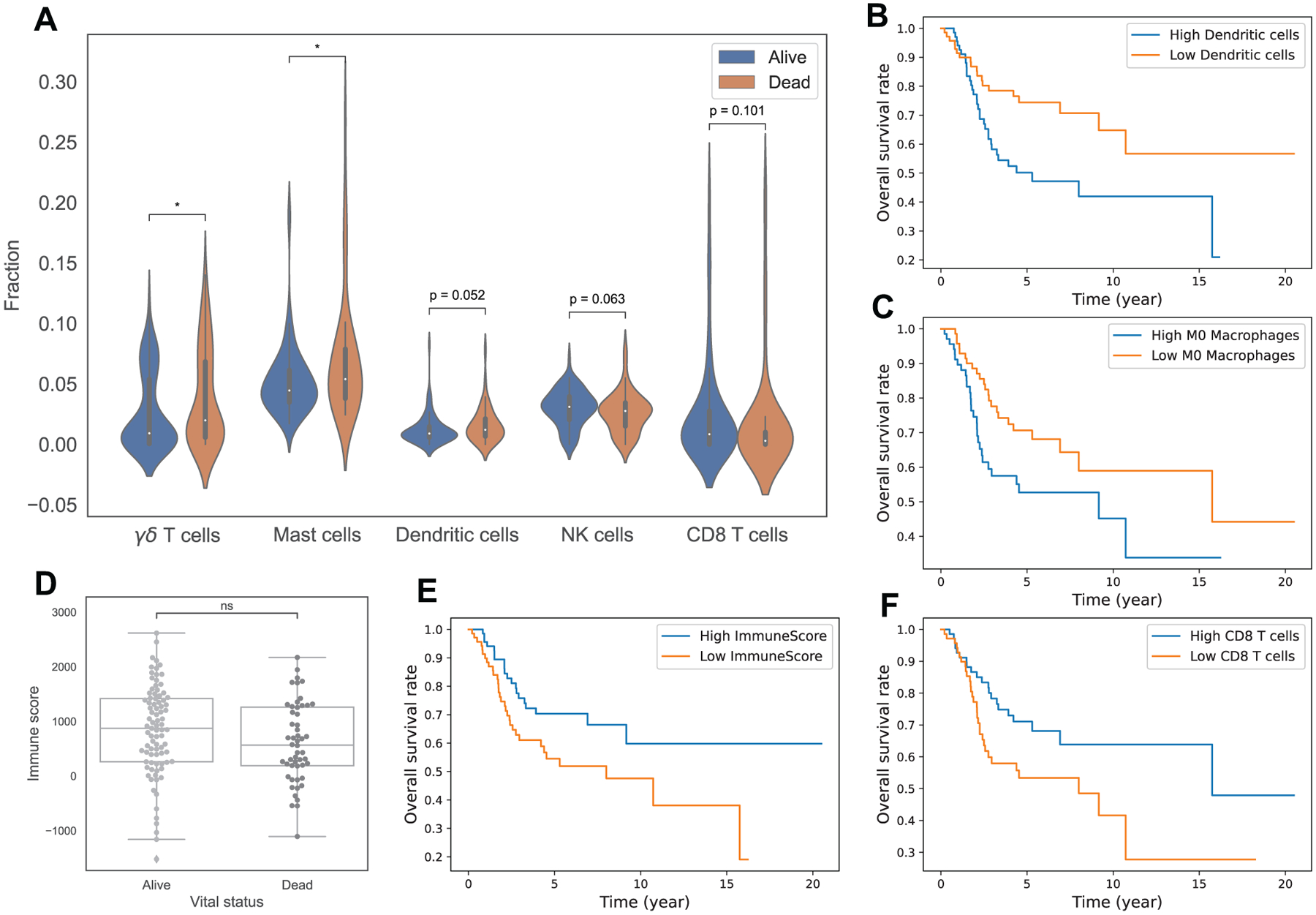
Relationship of immune infiltrations with survival in osteosarcoma. Sub-figure A displays violin plots of fractions of *γδ* T cells, Mast cells, Dendritic cells, NK cells, CD8 T cells between alive and dead patients. Sub-figures B, C, E, F show Kaplan-Meier curves of overall survival between 2 groups, B: high vs low Dendritic cells, C: high vs low M0 Macrophages, E: high vs low ESTIMATE immune score, F: high vs low CD8 T cells. Sub-figure D shows a boxplot of ESTIMATE immune scores between alive and dead patients. Note: asterisks indicate significant difference from Mann-Whitney-Wilcoxon test (ns: no significance, *: 0.01 < *p* ≤ 0.05, **: 0.001 < *p* ≤ 0.01, ***: 0.0001 < *p* ≤ 0.001, ****: *p* ≤ 0.0001).

Figure 3D indicates no clear difference in immune score between alive and dead patients at the last time of follow up. However, Kaplan-Meier curves of high and low immune score with a median cut-off reveal that the high immune score group has a better outcome (Figure 3E). The log rank test supports this observation with a p-value of 0.03. Thus, higher immune score is associated with better survival probability throughout time. This is consistent with the observation of outcome in the clusters. Cluster 2 has significantly lower immune score than cluster 1 and 3 (Figure 1F), with p-values of 5e–9 and 2.1e–5 from Mann-Whitney-Wilcoxon test, and accordingly worse overall survival probability over time than cluster 1 and 3 (Figure 2G). Cluster 1, with the highest average immune score among clusters (Figure 1F), even though has about the same proportion of dead patients at the last time of follow up as cluster 2 (Figure 2C), shows better survival time than cluster 2 (Figure 2G).

### 3.7. Relationship between immune infiltrates and survival outcome in osteosarcoma

The Mann-Whitney-Wilcoxon test shows that there is a significant difference in the level of *γδ* T cells and Mast cells between alive and dead patients at the last time of follow up, with p-values of 0.045 and 0.022, where dead patients are associated with higher percentages of *γδ* T cells and Mast cells than alive patients (Figure 3A). Dendritic cells, NK cells and CD8 T cells also seem to associate with survival status in osteosarcoma. We observe higher level of NK cells (p = 0.063), CD8 T cells (p = 0.1) and lower level of Dendritic cells (p = 0.052) in alive patients than in dead patients (Figure 3A).

Kaplan-Meier curves (Figure 3B, C, F) and the log rank test indicate an association between survival outcomes and levels of Dendritic cells, M0 Macrophages and CD8 T cells, with a p-value of 0.01, 0.04 and 0.04, respectively. Low Dendritic cells, low M0 Macrophages and high CD8 T cells are associated with better survival probability over time in osteosarcoma patients. This is again consistent with the outcome of the clusters where cluster 2, with the highest level of M0 Macrophages and lowest CD8 T cells, has the worst overall survival.

Overall, we found that *γδ* T cells, Mast cells, Dendritic cells, M0 Macrophages, NK cells and CD8 T cells have a relationship with the survival of osteosarcoma patients.

### 3.8. Association of immune infiltrates with other clinical features

**Figure 4.**
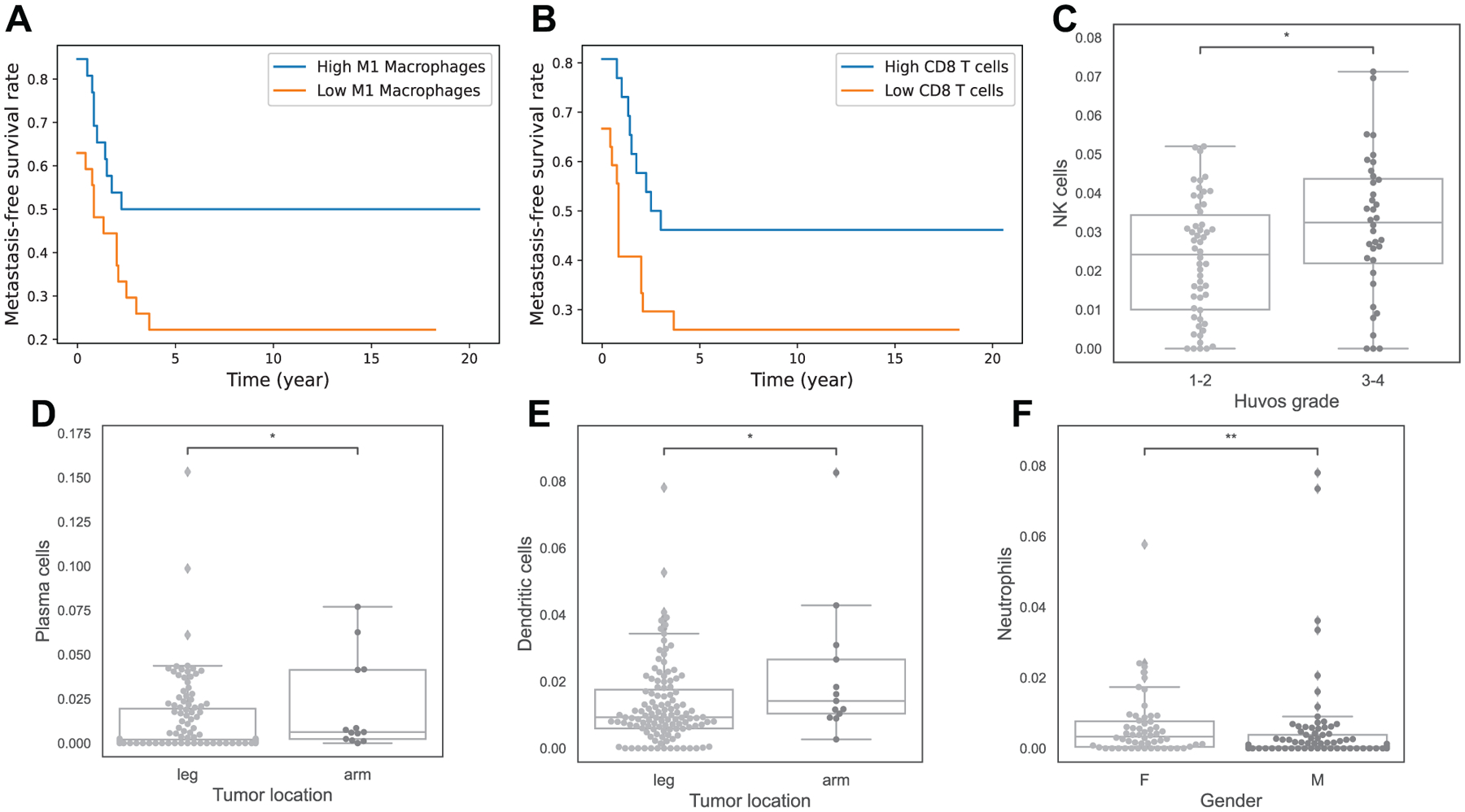
Association of immune infiltrations and other clinical features. Sub-figures A and B show Kaplan-Meier curves of metastasis-free-survival in cohort 1 between 2 groups, A: high vs low M1 Macrophages, B: high vs low CD8 T cells. Sub-figures C-F are boxplots to indicate relationship of immune infiltrates with Huvos grade (C), primary tumor location (D and E), and gender (F). Note: asterisks indicate significant difference from Mann-Whitney-Wilcoxon test (ns: no significance, *: 0.01 < *p* ≤ 0.05, **: 0.001 < *p* ≤ 0.01, ***: 0.0001 < *p* ≤ 0.001, ****: *p* ≤ 0.0001).

We see no significant relationship between age or metastasis at diagnosis and the frequencies of immune cells. However, we notice an association of M1 Macrophages and CD8 T cells’ frequencies to metastasis-free survival. High levels of M1 Macrophages and CD8 T cells are associated with better metastasis-free survival probability across time in osteosarcoma (Figure 4A, B), with p-values of 0.05 and 0.08 from the log-rank test, respectively. This means that patients with more M1 Macrophages and CD8 T cells are less likely to develop metastasis or die at any given time than patients with low percentage of these cells.

A relationship between some immune infiltrates and other clinical features of osteosarcoma has also been observed. Higher level of NK cells is associated with good response to chemotherapy (p = 0.035, Figure 4C). Patients with arm tumors have higher percentage of Plasma cells and Dendritic cells than patients with leg tumors (Figure 4D, E), with p-values of 0.046 and 0.033. Lastly, female patients are shown to have higher frequency of Neutrophils (Figure 4F), with p-value 0.0016.

### 3.9. Expression level of genes encoding PD-1, INF-γ, CTLA4, TNF, IL1-β, IGF1, IL-6 and RUNX2 are significantly different for some clusters

We use the gene expression values for some important proteins, and we analyze gene expression value of the proteins separately for cohort 1 and 2 because they have different data types: RNA-Seq and microarray, respectively.

**Figure 5.**
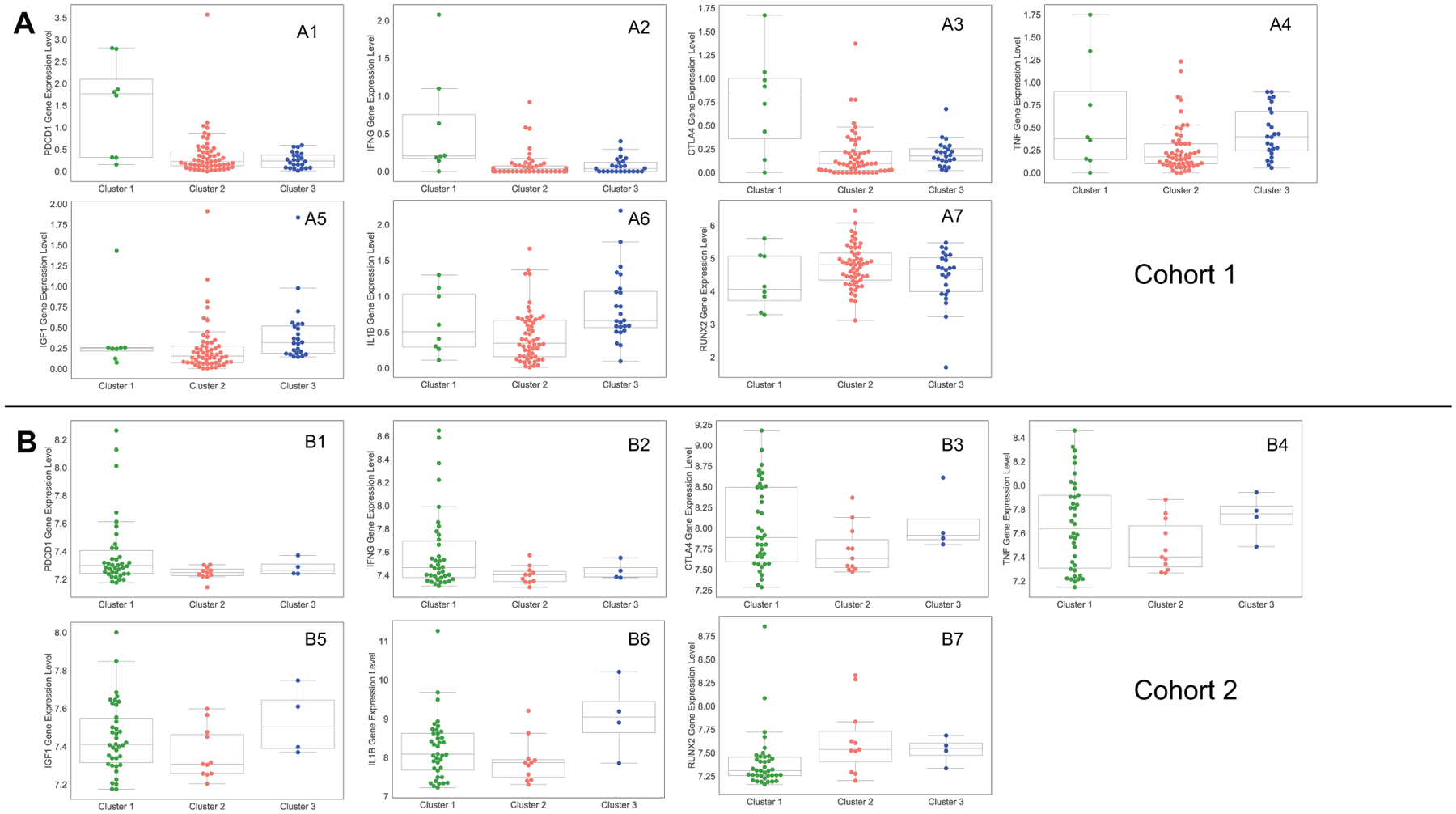
Gene expression values of important proteins in the clusters. Sub-figures (A1–A7) and (B1–B7) show the gene expression values that come from cohort 1 and cohort 2 data sets, respectively.

**Figure 6.**
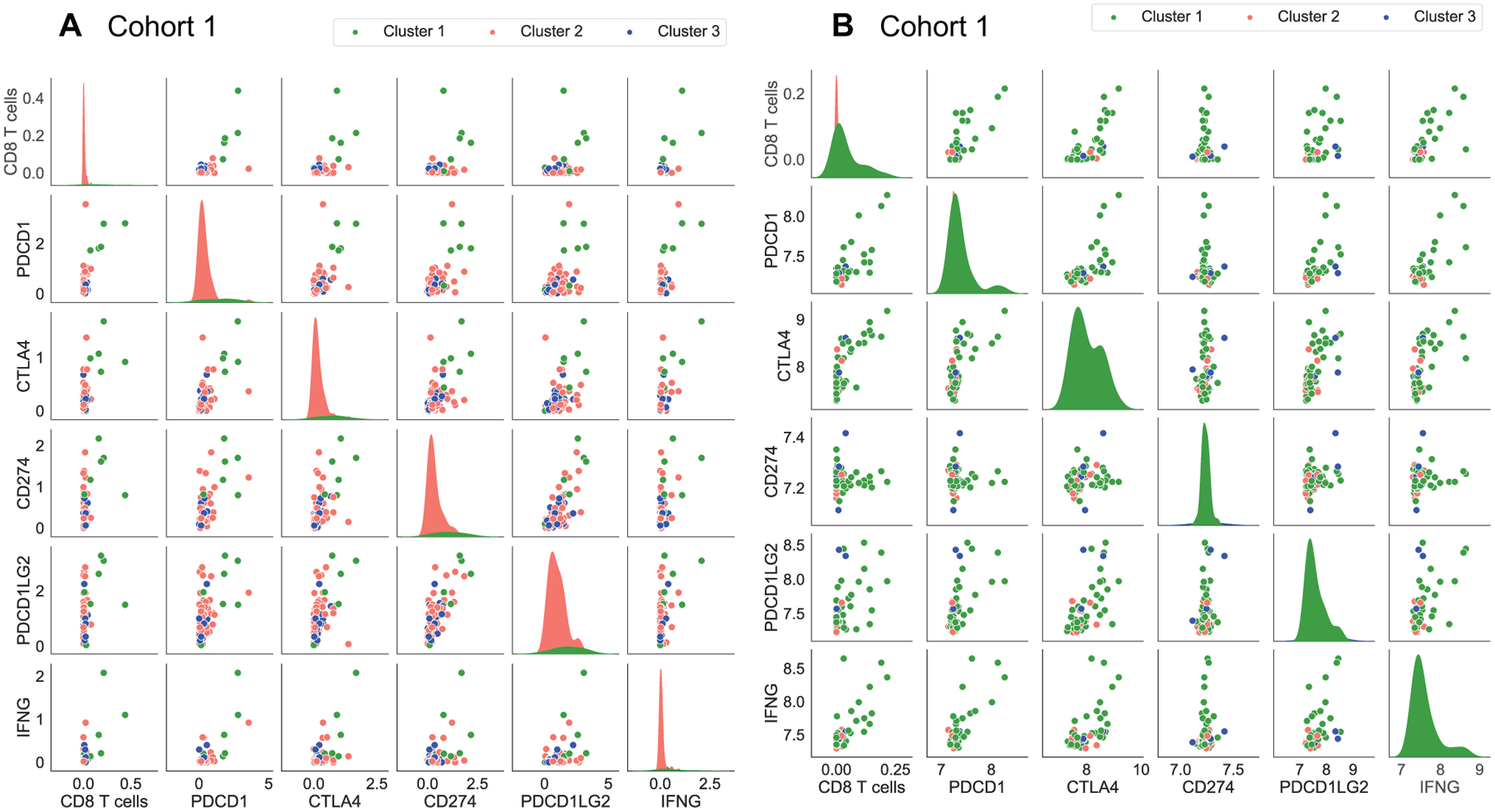
Correlation and distribution of important proteins in the clusters. Sub-figures A and B represent the cohort 1 and cohort 2 data sets respectively.

Programmed cell death protein 1 (PD-1) is a type of protein on T cells and cancer cells use it to bind with PD-1 ligand (PD-L1) and PD-2 ligand (PD-L2) to escape cell death by immune cells. There is a high correlation between PDCD1 gene, which encodes PD-1 protein, and CD8 T cells in both data sets with correlation coefficient of 0.70 and 0.77, respectively, and p-values less than 0.05 (Figure 6). As a result of this correlation, PDCD1 is the highest in cluster 1 (Figure 5A1, B1). Cluster 1 also has the highest expression of CTLA4 gene (Figure 5A3, B3) that encodes Cytotoxic T-Lymphocyte Associated Protein 4 (CTLA4), which is a member of immunoglobulin superfamily and has been found to significantly associate with the risk of osteosarcoma [38,39]. Moreover, we see that gene expression value of CTLA4 is significantly correlated with CD8 T cells in osteosarcoma tumors (Figure 6).

Interferon *γ* (INF-*γ*), encoded by IFNG gene, has antiviral, immunoregulatory, and anti-tumor properties in the immune system and is secreted by mostly T cells and NK cells [40]. Importantly, CD8 T cells frequency and PDCD1 gene expression are significantly correlated with IFNG gene expression so that cluster 1 has the highest level of IFNG compared the other clusters (Figure 5A2, B2). Beside these, we do not see any significant correlation between expression levels of CD274 and PDCD1LG2 genes, that encodes PD-L1 and PD-L2 respectively, with the expression levels of PDCD1 and IFNG, and the percentage of CD8 T cells in osteosarcoma tumors (Figure 6).

Tumor necrosis factor (TNF) is a cytokine that is mainly produced by Macrophages and has crucial roles in tumor development and tumor progression inducing apoptosis, necrosis, angiogenesis, immune cell activation, differentiation, and cell migration [41]. We notice that cluster 2 has the lowest TNF gene expression among clusters, while cluster 1 and 3 have roughly similar average expression of this gene (Figure 5A4, B4). In addition, we analyze gene IL1B that encodes cytokin protein Interleukin-1 beta (IL-1*β*), which is produced by activated Macrophages [42], and see that IL1B gene expression is the lowest in cluster 2 and the highest in cluster 3 (Figure 5A6, B6).

Beside these, Insulin-like growth factor 1 (IGF-1) is a hormone that has important function in the development and function of many tissues and it has been used as a diagnostic marker for osteosarcoma [43, 44]. Similar to TNF and IL1B genes expression, cluster 2 has the lowest amount of IGF1 among other clusters (Figure 5A5, B5). Furthermore, we examine RUNX2 oncogene that is associated with amplifications and it has been found to correlate to poor response to chemotherapy in osteosarcoma [45, 46]. In our analysis, cluster 1 has the lowest amount of RUNX2 gene and cluster 2 and 3 show almost similar expression of RUNX2 gene (Figure 5A7, B7).

## 4. Discussion

The findings from analyses using estimated immune infiltrations in osteosarcoma have varied among studies, perhaps due to the small number of osteosarcoma tumors with available gene expression data in the literature. In this study, we find that infiltration of CD8 T cells, NK cells and M1 Macrophages have a positive association with prognosis, while infiltration of *γδ* T cells, Mast cells, M0 Macrophages and Dendritic cells have a negative association with prognosis. Yu et al. [24] also illustrates that high level of CD8 T cells is a good prognosis in their survival analysis, and results from [26] and [28] indirectly suggest the positive prognostic value of CD8 T cells. Tang et al. [26] reports that CD8 T cells infiltration has a positive correlation with CXCR3 expression which is related to good prognosis. Khader et al. [28] shows that low-risk patients have high level of CD8 T cells and NK cells, which supports with our conclusion on these cells. In agreement with our findings on prognostic value of M1 Macrophages, Song et al [31] demonstrates that high level of M1 Macrophages is associated with good prognosis, while Zhang et al. [22] and Tang et al. [26] imply the same from their results. Our conclusion about M0 Macrophages aligns with the results from [22] and [26], but contradicts with the finding from [34] that abundance level of M0 Macrophages is positively correlated with survival.

The observable difference in outcomes between clusters are likely due to the relationship between immune infiltrates and prognosis in osteosarcoma, because we cluster the patients based on their immune composition. Our results indicate that cluster 2 has the worst outcome, while cluster 3 seems to have the best outcome among clusters. The main difference in immune composition between these two clusters is that cluster 2 has much higher percentage of M0 Macrophages, and lower percentage of CD8 T cells and M1 Macrophages than cluster 3. In general, we found that high levels of CD8 T cells and M1 Macrophages are associated with good prognosis, while a high level of M0 Macrophages correlates with poor prognosis in osteosarcoma. These results make sense because CD8 T cells are known to kill cancer cells directly [47, 48] and M1 Macrophages exhibit anti-tumor effects by producing cytokines that inhibit osteosarcoma growth [49]. These facts could also explain the observed differences between the outcomes of patients in clusters 2 and 3. Meanwhile, cluster 1 has worse outcome than cluster 3, but better outcome than cluster 2. This could be due to the fact that cluster 1 has both high level of immune cells associated with good prognosis such as CD8 T cells and M1 Macrophages, and high level of immune cells associated with poor prognosis according to our results such as *γδ* T cells and Mast cells. A high infiltration of mast cells has been associated with poor prognosis, low survival and increased metastasis in many cancers [50], while *γδ* T cells show dual effects on cancer growth [51]. Both mast cells and *γδ* T cells promote tumor development by supporting angiogenesis through angiogenic factors production [50, 51]. Mast cells also produce proteases, which lead to extracellular matrix degradation and tissue remodeling, and thus promote tumor growth [50]. *γδ* T cells have been reported to secrete TGF-*β* [51], which is a pro-tumor cytokine in osteosarcoma [52–54]. Overall, the clinical outcomes of the clusters are consistent with our findings and biological knowledge on prognostic values of immune cells in osteosarcoma.

On the other hand, we did not observe any difference in age or metastasis at diagnosis between clusters. This can be explained by the lack of correlation between immune infiltrates and these clinical variables, which suggests that the immune composition of the primary tumor has no effect on age or metastasis status at diagnosis.

Immune checkpoints have an important role in the immune system to prevent autoimmune diseases, but unfortunately they can allow immune tolerance against tumors. PD-1 and CTLA-4 are the main checkpoints that tumor cells use to block immune system [55–57]. Blocking PD-1 pathway has improved oncological survival of several patients with metastatic cancers, including melanoma, renal cell carcinoma, and colon cancer [58, 59]. Also, targeting CTLA-4 in patients with metastatic melanomas demonstrates significant development about overall survival [60].

It has been reported that osteosarcoma patients treated with an anti PD-1 drug, Pembrolizumab, show some improvement in disease progression [61]. Combination of PD-1 and CTLA-4 blockade therapy in bone sarcoma have shown better response compared to single checkpoint inhibitor therapy [62]. Note, patients in cluster 1 have the highest expression levels of IFNG, PDCD1 and CTLA-4 that are significantly correlated with CD8 T cells (Figures 5 and 6), and it has been shown that INF-*γ* increases the CD8 T cells expansion [63]. Thus, patients in cluster 1 might respond well to combination of PD-1 and CTLA-4 blockade therapies.

It has been suggested in several studies that bacteria are able to activate anti-tumor immune responses [64, 65]. In a study with combination of Bacillus Calmette-Guerin (BCG) injection and tumor vaccine, 18% of the patients remained alive and disease-free and it has been reported that bacterial vaccine caused increased level of immunoregulatory cytokines such as TNF-*α*, IFN-*γ*, and IL1-*β* that might be involved in inducing tumor regression [66]. As a result, bacterial vaccine and inactivated tumor cells injection can be thought of as a treatment that activates anti-tumor immune responses [47]. In our results, cluster 2 has the lowest amount of gene expression level of immunoregulatory cytokines TNF-*α*, IFN-*γ*, and IL1-*β* (Figure 5) so that tumors in this cluster might be treated with bacterial vaccine. Otherwise, targeting RUNX2 oncogene with chemotherapy is suggested as a new therapeutic approach to osteosarcoma patients in recent studies [46, 67] and cluster 2 has the highest amount of RUNX2 gene expression values compared to other clusters (Figure 5A7–B7) so with the help of further studies, tumors similar to those in cluster 2 also might be good candidates to treat with targeting RUNX2 in conjunction to standard chemotherapy.

Targeting tumor associated macrophages (TAM) is another alternative treatment method for osteosarcoma tumors and treatments that suppress M2 Macrophages phenotype or block the polarization of M1 Macrophages to M2 Macrophages have shown positive results in several studies [68–71]. Thus, tumors in cluster 3, which has the highest amount of M2 macrophages (Figure 1D) can be treated with targeting TAMs.

## Appendix

### A.1. CIBERSORTx B-mode algorithm

CIBERSORTx is the latest version of CIBERSORT, which has been the most popular deconvolution method up until now. Both algorithms are based on the following model:

y=Xβ

where *y* is the vector of gene expression values of the bulk tumor, called mixture data, *X* is the signature matrix where each column is a vector of gene expression values of a cell type, and *β* is the vector of estimated cell fractions.

CIBERSORT solves for *β* using a machine learning technique called nu-Support Vector Machine (*v*-SVR). Specifically, CIBERSORT finds *β* by minimizing the objective function:

$$\frac{1}{2}\|\beta {\|}^{2}+C\left(v\epsilon +\frac{1}{N}\overset{N}{\underset{i=1}{\sum }}\left(\xi_{i}+\xi_{i}^{*}\right)\right)$$

s.t. $y_{i}-{\hat{y}}_{i}\leq \epsilon +\xi_{i}$ and ${\hat{y}}_{i}-y_{i}\leq \epsilon +\xi_{i}^{*}$ and$\xi_{i},\;\xi_{i}^{*}\geq 0$.

Here *ϵ* is the margin of error, *v* is a model hyperparameter between 0 and 1, *ŷ*_*i*_ is the predicted value of *y*_*i*_, and $\left(\xi_{i}+\xi_{i}^{*}\right)$ is the absolute error of *ŷ*_*i*_ that lie outside margin of error *ϵ* from *y*_*i*_. By minimizing this objective function, CIBERSORT finds estimated fractions $\hat{\beta }$ that result in the smallest absolute error between observed and predicted mixture data (*y* and *ŷ*) where a margin of error *ϵ* is tolerated. After this optimization step, all negative coefficients in the estimated $\hat{\beta }$ are set to 0 and the coefficients are normalized to sum to 1 because $\hat{\beta }$ represents cell fractions.

CIBERSORTx extends CIBERSORT by adding batch correction to adjust the potential cross-platform variations between mixture data *y* and signature matrix *X*. The algorithm of CIBERSORTx B-mode works as follows:

1. Given signature matrix *X* and mixture data *y*, obtain estimated cell fractions $\hat{\beta }$ from CIBERSORT
2. Compute estimated mixture data *ŷ* as *ŷ* = *X$\hat{\beta }$*
3. Use Combat, a well-known batch correction method, to adjust the technical variations between *y* and *ŷ*, outputing adjusted mixture data *y*_*ad*_*j*
4. Obtain final estimated cell fractions from *X* and *y*_*ad*_*j* using CIBERSORT

### A.2. ESTIMATE algorithm for Immune Score

Given an immune gene set, which is a set of genes associated with the quantity of immune infiltrations, ESTIMATE uses single sample gene set enrichment analysis (ssGSEA) to compute the Immune Score from gene expression data of bulk tumor. A high Immune Score represents a high level of infiltrating immune cells in the tumor tissue. ssGSEA algorithm in ESTIMATE works as follows:

1. Order gene expression data of bulk tumor from highest to lowest and replace gene expression values with their ranks
2. For each gene *i*, compute the following:
  $$P_{G}^{w}(G,y,i)=\frac{\underset{r_{j}\in G,j\leq i}{\sum }{\left|r_{j}\right|}^{\alpha }}{\underset{r_{j}\in G}{\sum }{\left|r_{j}\right|}^{\alpha }},$$
  $$P_{NG}(G,y,i)=\underset{r_{j}\notin G,j\leq i}{\sum }\frac{1}{\left(N-N_{G}\right)},$$
  where *G* is the given immune gene set which has *N*_*G*_ genes, *y* is the gene expression data of bulk tumor which has *N* genes, *r*_*j*_ is the rank of gene *j* and *α* is a hyperparameter between 0 and 1.
3. The Immune Score of sample *y* given immune gene set *G* is:
  $$IS(G,y)=\overset{N}{\underset{i=1}{\sum }}\left[P_{G}^{w}(G,y,i)-P_{NG}(G,y,i)\right].$$

### A.3. K-means clustering algorithm

Given a number of clusters *k*, K-means clustering works as follows:

1. Randomly choose *k* data points as centroids
2. Assign each data point to its closest centroid, resulting in *k* clusters based on *k* centroids
3. Compute a new centroid for each cluster by taking average of data points that belong to that cluster
4. Repeat step 2 and 3 until the centroids no longer change
